# The Impact of a Psychological Skills Training and Mindfulness-Based Intervention on the Mental Toughness, Competitive Anxiety, and Coping Skills of Futsal Players—A Longitudinal Convergent Mixed-Methods Design

**DOI:** 10.3390/sports11090162

**Published:** 2023-08-29

**Authors:** Daniel Vella-Fondacaro, Stephanie Romano-Smith

**Affiliations:** 1 Department of Psychiatry, Mental Health Services Malta, ATD 9033 Attard, Malta; 2 Faculty of Medicine and Surgery, University of Malta, MSD 2080 Msida, Malta; 3 School of Health, Science and Wellbeing, Staffordshire University, Stoke-on-Trent ST4 2DF, UK; stephanie.romanosmith@staffs.ac.uk

**Keywords:** futsal, psychological, intervention, anxiety, mixed methods, experience

## Abstract

Despite the sport’s popularity, there is a paucity in futsal psychological interventional research. This study analysed the impact of a ten-session psychological skills training and mindfulness-based intervention (PSTMI) on the mental toughness, competitive anxiety, and athletic coping skills of national league futsal players (n = 13). It also analysed whether these variables were predicted by playing experience. Pre-/post-intervention questionnaires were filled in and analysed (Competitive State Anxiety Inventory-2, Sport Mental Toughness Questionnaire, and Athletic Coping Skills Inventory-28). Semi-structured interviews were also conducted with seven athletes; quantitative and qualitative data were integrated in a convergent parallel mixed-methods design. Results revealed significant medium-to-large improvements in mental toughness, cognitive anxiety, and coping skills following the PSTMI. Years of playing experience positively and significantly predicted better self-confidence and coping skills. Thematic analysis generated five themes: (1) post-intervention enhancement in athletic performance and well-being; (2) the non-athletic commitments of futsal players; (3) diverse views on how to improve the intervention; (4) instilling social identity through sport psychology sessions; and (5) the impact of years of experience on skill learning. Results mirrored those from other sporting disciplines. The PSTMI was well-received and equipped athletes with beneficial psychological skills, stressing the need for more sport psychology resources in futsal.

## 1. Introduction

Mental training provides athletes with the skills to better deal with challenges during competition [1]. Previous research has reported a reciprocal and bidirectional relationship between mental health and sport performance [2,3,4]. Despite its empirical support, this bidirectional mental health model [5,6] has had its criticisms [7], including methodological shortcomings [8], and the proposal of alternative models. Such a model is the Iceberg Profile, which depicts a visual representation of different emotional states (tension, depression, anger, vigor, fatigue, and confusion) and their impact on athletic preparation and performance [9]. Another model is the Ecological Systems Model, which divides the elite athlete’s mental health into the athlete (e.g., coping skills and personality), microsystem (e.g., coaches, relatives, and friends), exosystem (e.g., individual sport), and the macrosystem (sporting environment and media) [10]. Alternatively, the Individual Zones of Optimal Functioning (IZOF) model suggests that athletes perform best when they are at their optimal level of anxiety [7]. Despite their differences, such models draw attention to the contribution of psychological factors in athletic success.

Psychological skills training (PST) consists of psycho-educational sessions to train a set of skills, namely motor imagery, arousal regulation, self-talk, and goal setting [11]. Mindfulness-based interventions (MI) focus on techniques such as awareness and non-judgmental acceptance in the form of mindfulness exercises [12]. Despite being fundamentally different, these two distinct interventions both aim to equip athletes with psychological skills and improve their mental state and performance. Sport research on the topic is extensive [13], suggesting improvements in athletes’ mental toughness [14], cognitive anxiety [15], and coping skills [16,17], among others, following PST and/or MI. Research has also reported reduced anxiety levels in more experienced athletes [18,19].

Despite becoming increasingly popular [20,21], psychological research in futsal is lacking, with most research being performance-based and cross-sectional, limiting the possibility of drawing causal inferences [22,23]. Furthermore, Yeemin et al. (2016) carried out a systematic review to analyse 23 sport psychology studies in futsal. The authors identified a lack of mixed-methods research, with the first article published in 2008. This lack of research can be attributed to the low financial interest in the game [24]. Futsal has often been used to develop athletes for high-performance football [25], sidelining futsal research [26]. As with other sports, mental training with futsal athletes has been associated with an improved performance [27,28].

This study addressed the lack of research within this area by carrying out a pretest–posttest interventional study using a convergent mixed-methods design, being the first known mixed-methods psychological study in futsal. The mixed-methods design merged quantitative and qualitative data: quantitative data served to help provide better generalizability of the findings, while qualitative data provided a deeper exploration into the research question. Previous interventional studies in sport research focused on either PST or MI, or compared the two [29,30]. This study innovatively looked at a combination of PST and MI sessions as one joint psychological skills training and mindfulness-based intervention (PSTMI). This aimed to widen the psychological skill repertoire covered during the intervention. The skills covered in this intervention included goal setting, motor imagery, arousal regulation, mindfulness exercises, and self-talk. All these psychological skills were attributed with better attention and emotional control in athletes, better functional control after failure, and better overall performance-relevant psychological factors [29]. Furthermore, self-talk training has been associated with better levels of self-confidence and less somatic state anxiety in athletes [15]. Mindfulness training has also been associated with improved coping styles [17]. While acknowledging the overlapping nature between PST and MI in arousal regulation, the differences between the two were appreciated. However, given the targeted outcomes, a combination of both approaches was deemed more appropriate for the team’s needs, training style, and preference.

This study aimed to investigate whether a PSTMI helped improve the mental toughness, competitive anxiety (cognitive anxiety, somatic anxiety, and self-confidence domains), and coping skills of futsal players. While acknowledging that some anxiety is not always necessarily a negative construct, the anxiety levels analysed in this study’s questionnaires were those causing the athletes considerable distress and impeding their performance. Furthermore, better athletic coping skills have been associated with a better overall performance [16]. Mental toughness has been defined as the ability of an athlete to rebound from failure, face adversity, and cope with pressure [14].

Lower athletic experience has been associated with higher competitive anxiety [15]. The PSTMI worked on several psychological variables, including competitive anxiety. Therefore, this study also aimed to evaluate whether years of experience in futsal significantly predicted the competitive anxiety, mental toughness, and coping skills of futsal players within the same team.

## 2. Materials and Methods

### 2.1. Participants

Thirteen male futsal players were recruited by convenience sampling for a ten-session PSTMI. The participants played for a national futsal league team in Malta. The age range (19–29 years, mean = 26.26 years) encompassed the ‘mastery’ stage of athletic transition [31]. The athletes had different levels of futsal experience (range = 1–10 years; mean = 5.15 years). Eleven athletes were Maltese, one was Spanish, and one was Libyan. Seven of the athletes were randomly selected for qualitative interviews, until thematic saturation was established. This ensured that the qualitative results obtained were robust and as accurate as possible. All data were stored anonymized in a password-protected spreadsheet within an encrypted computer.

### 2.2. Design

**Figure 1:**
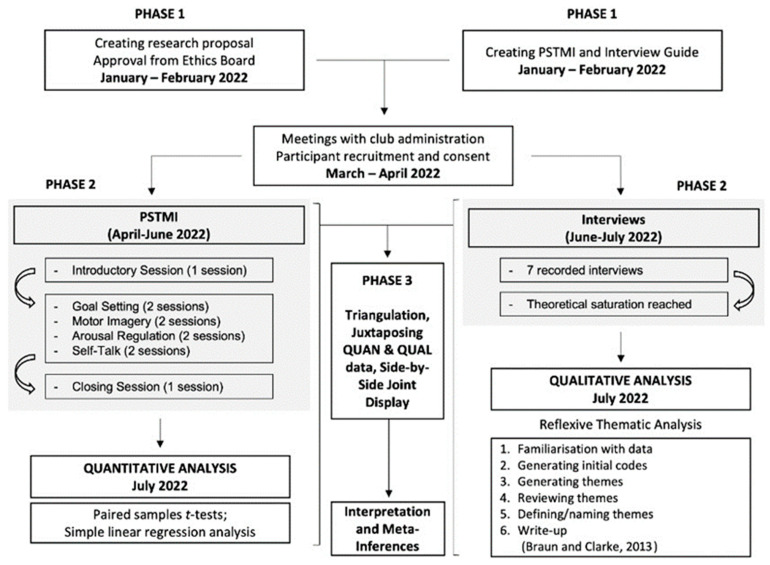
A Flowchart Describing the Research Method.

This study employed a convergent, parallel mixed-methods design (Figure 1), utilizing both quantitative and qualitative methods. Therefore, the study’s epistemological stance was within a pragmatic paradigm, integrating postpositivist and interpretivist frameworks [32]. The quantitative (pre-/post-intervention scores) and qualitative (interviews) strands of the research were actioned and analysed independently. Integration of quantitative and qualitative data [33] was then carried out for overall interpretation [34].

### 2.3. Data Collection

Pre- and post-intervention scores were collected using the Sports Mental Toughness Questionnaire (SMTQ) for mental toughness [35], the Competitive State Anxiety Inventory-2 (CSAI-2) for competitive anxiety [36], and the Athletic Coping Skills Inventory-28 (ACSI-28) for coping skills [37]. The SMTQ is a 14-item self-report questionnaire assessing mental toughness in sport, with responses rated on a 4-point Likert scale ranging from “not at all true” (score = 1) to “very true” (score = 4). It contains 3 subscales: confidence (6 items), constancy (4 items), and control (4 items), with a total score out of 56 [35]. The CSAI-2 is a self-reported, 27-item questionnaire assessing cognitive anxiety (CSAI-2-CA), somatic anxiety (CSAI-2-SA), and self-confidence (CSAI-2-SC), with 9 items each (total score = 36). Every item is on a 4-point Likert scale ranging from “not at all” to “very much so”. The domains are assessed separately, and therefore, there is no total score for the questionnaire [36]. The ACSI-28 is a self-reported 28-item questionnaire used to assess the level of athletic coping skills, with responses rated on a 4-point Likert scale ranging from “almost never” (score = 0) to “almost always” (score = 3). The ACSI-28 is made up of seven subscales (coping with adversity, peaking under pressure, goal setting/mental preparation, concentration, freedom from worry, confidence/achievement motivation, and coachability). It has a total score of 84 [37]. Therefore, whereas the CSAI-2 was used for its subscales in our results, the SMTQ and ACSI-28 final scores were used, as the latter two can be added up. Instructional manipulation checks (‘trap questions’) were included to minimize response bias and improve reliability [38]. Trap questions were included randomly within the questionnaire and included obvious and non-relevant questions, to ensure that the participants were attentive and followed the appropriate instructions.

An eight-component semi-structured interview guide focused on the athletes’ perspectives on the PSTMI sessions. It also addressed the intervention’s impact on the athletes’ mental toughness, competitive anxiety, and coping skills. Examples of such questions were: How did you feel that this intervention went? What do you feel was helpful? What do you feel was less helpful or distressing? Do you feel less/more anxious when you think about competitions, and how so? A semi-structured approach was chosen to facilitate an open discussion, allowing athletes to elaborate on sensitive topics which were not predetermined by the researcher [39]. The guide and quantitative questionnaires were tested for readability and found to be age appropriate.

### 2.4. Procedure

#### 2.4.1. Ten-Session PSTMI

**Figure 2:**
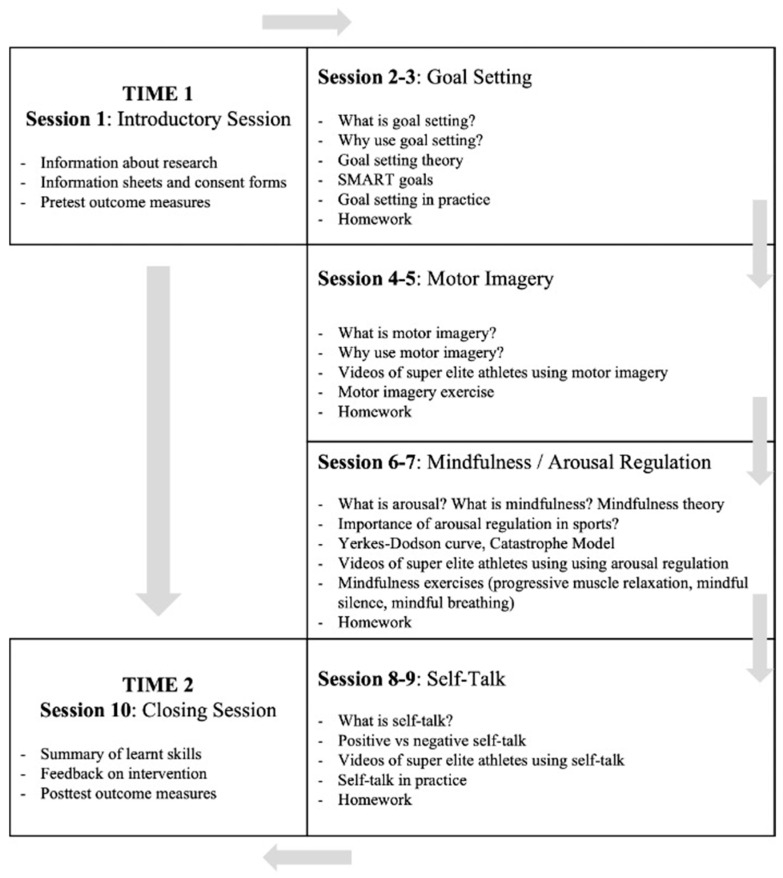
A Flowchart Describing the PSTMI.

The ten-session intervention (PSTMI, Figure 2) included an introductory session, eight skill sessions (goal setting, motor imagery, mindfulness/arousal regulation, and self-talk), and a closing session. Goal setting and motor imagery sessions were planned for the first half of the program as they were carried out during the preparation and early phases of the national cup competition. Conversely, mindfulness/arousal regulation sessions and self-talk sessions were planned for the second half of the PSTMI during the more challenging games of the competition. The PST and MI sessions were adapted from previous research [29,40,41,42] and merged into a more inclusive PSTMI, acknowledging the overlap between mindfulness and arousal regulation [16]. Furthermore, both PST and MI have been reported to improve emotional control in athletes, and both can be applied successfully as they possess “shared effects” [29]. While PST focused more on psychological skills, mindfulness sessions focused on exercises aimed at “letting go”, using a non-judgmental approach, and relaxation techniques, such as progressive muscle relaxation and mindful silence and observation. The ten sessions spanned the entirety of a national futsal knockout competition over an eight-week period and were carried out by a warranted psychiatrist doing a master’s degree in Sport and Exercise Psychology (main researcher). Sessions were carried out once weekly.

Each session lasted thirty minutes, which covered the skill in required detail, while avoiding reductions in sustained attention levels associated with longer sessions [43]. The intervention was carried out as a group, supporting team cohesion [44]. The sessions were held at the Malta National Sports School, the official venue for league and cup matches.

The introductory session informed athletes of the research, and consent was obtained. All 13 athletes agreed to participate. Baseline pre-intervention questionnaires were distributed during the introductory session. The subsequent eight sessions covered goal setting, motor imagery, arousal regulation/mindfulness, and self-talk, respectively. During these sessions, the skill was explained using screen presentations, videos, and group discussions. Athletes were reminded to practice the skills between sessions, during practice and competitive matches. The practiced skills were then discussed during the start of the next session and the athletes shared their experience with each other. The closing session was held online as the futsal season had ended. Feedback was gathered, and post-intervention questionnaires were distributed online.

#### 2.4.2. Qualitative Interviews

Seven semi-structured interviews were carried out, each lasting around 60 min. The seven athletes were chosen by lot. Four interviews were held in person and three were held online due to preference. Seven interviews were sufficient to reach theoretical saturation, making further interviews unnecessary [45], which was in line with previous research [46,47]. Thematic analysis (TA) guidelines [48] reported that 6–10 interviews are recommended for such studies. Since all participants understood the English language, which is an official language in Malta [49], all interviews were carried out in English.

### 2.5. Data Analysis

IBM SPSS Statistics 28.0 (IBM Corp., Armonk, NY, USA) was used for statistical analysis. The data were screened for parametric assumptions, and parametric tests were used. The Shapiro–Wilk test [50] confirmed a normal distribution of data variables. The Mahalanobis’ distance test [51] and *z*-scores were used to exclude outliers. Homoscedasticity was confirmed using standardized residual scatterplots and by using the *F*-test for the *R*^2^ statistic as an approximation to the Breusch–Pagan test [52].

Paired samples *t*-tests analysed pre-intervention (Time 1) and post-intervention (Time 2) changes in dependent variables, by comparing the pretest/posttest data mean for the same group of participants, for every independent variable. Hedges’ *g* effect size [53] was used due to the small sample [54]. Effect sizes of 0.2, 0.5, and 0.8 were used to interpret small, medium, and large effects, respectively [55,56]. Two-tailed hypothesis testing was used to consider the possibility that such a psychological intervention can cause worsening in psychological variables. Furthermore, simple linear regression analysis was used to predict response variables from a predictor variable. Therefore, it analysed how years of futsal experience predicted variances in mental toughness, cognitive anxiety, somatic anxiety, self-confidence, and coping skills. Statistical significance was set at *p* < 0.05.

Qualitative interviews were recorded verbatim, carefully transcribed, and analysed using reflexive thematic analysis (RTA) [57]. As per RTA guidelines [57], the researchers: (i) familiarized themselves with the data, (ii) coded the data, (iii) generated initial themes, (iv) reviewed, and developed themes, (v) refined, and defined final themes, and (vi) produced the report. Manual coding of data was carried out. In addition to these steps, which were also included in the introduction of TA as a concept [48], RTA included an element of reflexivity. Therefore, during theme formation, the researchers kept an open mind and reflected on potential assumptions, subjectivity, and interpretations to flexibly create themes based on data and reflexive practice. A convergent mixed-methods analysis in the form of a between-method triangulation [58] was utilized. This involved the juxtaposition and integration of quantitative and qualitative results using a side-by-side joint display to generate meta-inferences [59,60].

### 2.6. Ethical Information

Ethical approval was obtained from the Staffordshire University Research Ethics Committee on the 17 February 2022. The research was approved by the Futsal Club directorship on the 28 March 2022. Verbal and written informed consent were obtained from all participants, and an information sheet was provided with details of freedom to opt out from the study at any point, confidentiality, etc. All data were stored in a password-protected spreadsheet on an encrypted computer.

## 3. Results

### 3.1. Quantitative Analysis

#### 3.1.1. Changes in Pre- and Post-Intervention Outcome Measures

Cronbach’s alpha (α) was used to measure the internal consistency for all pre- and post-intervention outcome measure results (α = 0.78–0.94). The pre- (α = 0.78) and post-intervention (α = 0.81) SMTQ scores consisted of 14 items. The CSAI-2 included the pre- (α = 0.92) and post-intervention (α = 0.89) cognitive state anxiety subscale scores, the pre- (α = 0.91) and post-intervention (α = 0.90) somatic state anxiety subscale scores, and the pre- (α = 0.90) and post-intervention (α = 0.94) self-confidence subscale scores, consisting of nine items per subscale. Finally, the pre- (α = 0.93) and the post-intervention (α = 0.92) ACSI-28 scores consisted of 28 items.

Results (Table 1) revealed a significant improvement in mental toughness (significant increase in SMTQ scores) from Time 1 (*M* = 37.85; *SD* = 5.956) to Time 2 (*M* = 41.85; *SD* = 6.581), *t* (12) = −2.996, *p* = 0.011, with a medium–large effect size (Hedges’ *g* = 0.778). There was a significant improvement in cognitive anxiety (significant decrease in CSAI-2-CA scores) from Time 1 (*M* = 23.85; *SD* = 6.026) to Time 2 (*M* = 21.00; *SD* = 5.492), *t* (12) = 3.008, *p* = 0.011, with a medium–large effect size (Hedges’ *g* = 0.781). There was also a significant improvement in coping skills (significant increase in ACSI-28 scores) from Time 1 (*M* = 45.38; *SD* = 14.655) to Time 2 (*M* = 50.62; *SD* = 13.805), *t* (12) = −2.775, *p* = 0.017, with a medium–large effect size (Hedge’s *g* = 0.720).

Small non-significant improvements were noted in somatic anxiety (non-significant decrease in CSAI-2-SA scores: *t* (12) = 0.783, *p* = 0.449; Hedges’ *g* = 0.203) and self-confidence (non-significant increase in CSAI-2-SC scores: *t* (12) = −0.581, *p* = 0.572; Hedges’ *g* = 0.151).

#### 3.1.2. Years of Experience Predicting Difference in Post-Intervention Outcome Measures

Two significant models emerged (Table 2). Years of experience positively and significantly predicted self-confidence: *F*(1, 11) = 7.971, *p* = 0.017, Beta = 0.648 (predicted 42.0% of variance, *R*^2^ = 0.420). Years of experience also positively and significantly predicted coping skills levels: *F*(1, 11) = 7.897, *p* = 0.017, Beta = 0.646 (predicted 41.8% of variance, *R*^2^ = 0.418). The regression models for mental toughness, cognitive anxiety, and somatic anxiety were non-significant.

### 3.2. Reflexive Thematic Analysis

**Figure 3:**
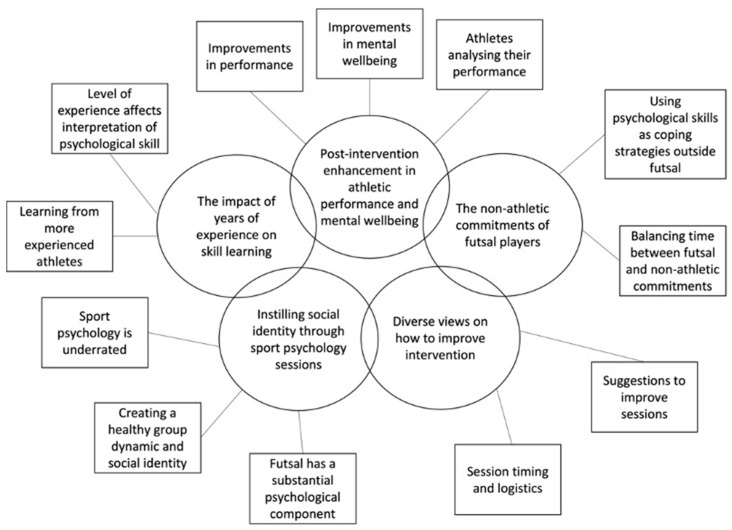
Thematic Map Including Themes (Circles) and Subthemes (Rectangles).

RTA revealed 5 themes and 12 subthemes (Figure 3).

#### 3.2.1. Theme 1: Post-Intervention Enhancement in Athletic Performance and Mental Wellbeing

All interviewed athletes felt that the PSTMI sessions helped their mental wellbeing. Some (n = 3) felt that sessions were “*very informative and very applicable*” (P7), serving as a period of realization: “*It helped me realize that I do actually need this*.” (P3). Athletes (n = 4) described having “good mental preparation” (P2) and a confidence boost following the sessions: “…*if we believe in it, we can beat anyone.*” (P11). Athletes (n = 5) started feeling less anxious: “*About two months ago, when you started the sessions, my anxiety was… quite high. But then, I was able to think better…*” (P12).

#### 3.2.2. Theme 2: The Impact of Years of Experience on Skill Learning

Most athletes (n = 6) felt that having more futsal experience helped with psychological skill learning and coping strategies: *“You’d know the feelings and anxiety more, you would have experienced it more.”* (P3). Nevertheless, athletes felt that psychological sessions should start early on: *“…getting professional guidance from day one is much better…”* (P7). Showing videos of experienced superelite athletes was helpful: *“This is something that’s routinely done and it’s helping top players achieve and maximize their performance.”* (P7).

#### 3.2.3. Theme 3: Instilling Social Identity through Sport Psychology Sessions

All interviewed athletes felt that sport psychology is underrated and was something that they *“had never experienced”* (P8), partially due to mental health stigma: *“…people think that it makes you look weak if you speak about something like that”* (P11). Athletes (n = 6) believed that PSTMI sessions helped shun this “*taboo*” (P11) by establishing a healthy team dynamic. *“There’s a very good dressing room environment, especially compared to other teams.”* (P7). This helped develop a club social identity: *“…we, university, are a top team, so we can’t play like others.”* (P12). Some participants (n = 3) felt that holding the sessions as a team enhanced their spirit of togetherness: *“…helped us a lot because we were bouncing ideas off each other.”* (P2).

#### 3.2.4. Theme 4: Diverse Views on how to Improve the Intervention

Most athletes (n = 5) felt that the intervention was carried out during a *“crucial period of time”* (P7), which helped retain the learnt psychological skills during competition: *“While if you’d do it the night before the game, some things are still fresh in your mind…” (P2)*. Short 30 min sessions helped participants maintain their attention span: *“If the sessions are long, you tend to lose concentration…”* (P11).

Athletes suggested using videos of local athletes, as these are more relatable: *“But if it’s done by someone who is living in the same conditions as you, maybe more people will start believing in it.”* (P7). Some (n = 2) suggested online sessions and small-group breakout rooms (n = 2) as *“participants would be more vocal when in a smaller group”* (P5).

#### 3.2.5. Theme 5: The Non-Athletic Commitments of Futsal Players

All interviewed athletes spoke about difficulties with balancing their athletic and non-athletic commitments: *“I went into the season like hardly thinking about futsal because I was finishing my thesis.”* (P3). Others, referred to work commitments: *“…so maybe sometimes you’d be tired for your session, because you would have had a day of work…”* (P8). Most athletes (n = 6) felt that learned coping skills can be used to minimize competitive anxiety in non-sporting areas: “*I think even before something like a work interview, they might even come in handy.”* (P5).

### 3.3. Integrating Quantitative and Qualitative Data

The integrative analysis, organized by research questions in a side-by-side joint display, generated three meta-inferences: (1) participants were very interested in the sessions, appreciated the content, and agreed with the PSTMI format, (2) the PSTMI had a positive impact on the participants’ levels of mental toughness, cognitive anxiety, and coping skills, by equipping them with helpful and practical psychological skills, and (3) more experience in the sport helped improve self-confidence and the use of coping skills to minimize competitive anxiety. No points of divergence were identified.

## 4. Discussion

### 4.1. The Impact of the Intervention on Mental Health Variables

To the researcher’s knowledge, this was the first psychological, mixed-methods study on futsal athletes, and the first futsal interventional study in Malta. We analysed the impact of a PSTMI on mental health variables in futsal athletes, which generated significant improvements in mental toughness, cognitive anxiety, and coping skills. Similar research in futsal is limited; however, previous studies in other sporting disciplines have reported similar results. Due to the lack of such research in futsal, psychological interventional research from other sporting disciplines was used for result comparison. A meta-analysis [61] reported medium-to-large improvements on cognitive anxiety (*g* = 0.54) following a psychological intervention, supporting the medium-to-large effects obtained from our research (*g* = 0.781, *p* = 0.011). However, this meta-analysis also reported medium-to-large improvements in self-confidence (*g* = 0.55), contrasting with the non-significant results from our research, possibly due to the team being viewed as the year’s “underdogs”, reducing their confidence levels [62]. Furthermore, in their meta-analysis, Ong and Chua (2020) reported that the interventional impact might be greater for athletes of a higher competitive level than those with lower competitive levels. Therefore, one might hypothesize that since most of the athletes in our intervention were contracted on an amateur level, this might have limited the intervention’s overall impact on the athletes’ self-confidence. The significant medium-to-large improvements in mental toughness (*g* = 0.778, *p* = 0.011) and coping skills (*g* = 0.720, *p* = 0.017) obtained from our study also mirrored previous research in other sports [29,63].

A systematic review of thirty meta-analyses [13] had reported improvements in athletic performance with improved confidence and cohesion following sport psychology interventions. The quantitative and qualitative data obtained from our study replicated these findings. Lochbaum et al. (2022) also suggested that cognitive anxiety had a small negative effect on athletic performance. This further highlights the need for interventions such as the PSTMI to improve the athletes’ levels of self-confidence and cognitive anxiety, among others, to improve performance and the general athletic experience.

Interventional futsal research to date has focused mainly on performance enhancement rather than mental wellbeing [27,64]. This lack of psychological awareness in futsal was reflected in our study’s qualitative results. Despite being the “underdogs”, the team chosen for our study won the knockout tournament (E&L Futsal Trophy); the final being against a team which was unbeaten for four seasons. Interviewed athletes suggested that the PSTMI helped them reach this successful milestone. Qualitative results also suggested that the PSTMI sessions helped the team adopt a social identity approach [65] towards the club, harboring a better spirit of togetherness and self-categorizing [66,67] their team as a winning team.

The post-intervention performance enhancement reported by the athletes may be attributed to the significant improvements in mental wellbeing [68,69] and the club’s social identity [70] following the intervention. Reflecting on the qualitative data obtained from the semi-structured interviews, it was evident that the athletes showed a sense of self-pride and motivation following the end of the season. While acknowledging that these positive responses might have been biased after winning the cup, all the interviewed athletes showed a high level of comradery towards each other. The qualitative association between the PSTMI and improved levels of performance described during the interviews has been reported in previous research [68].

### 4.2. The Impact of Level of Experience on Mental Health Variables

This research analysed whether the athletes’ years of experience significantly predicted the levels of mental toughness, cognitive anxiety, somatic anxiety, self-confidence, and coping skills. Post-intervention scores were used as response variables, as these included post-intervention improvements.

No similar futsal research was identified in the literature. Results from our research reported that years of experience positively and significantly predicted self-confidence (Beta = 0.648, *R*^2^ = 0.420, *p* = 0.017) and coping skills (Beta = 0.646, *R*^2^ = 0.418, *p* = 0.017), echoing previous research from other sporting disciplines [71,72]. One possible explanation is the improved skillset associated with more experienced athletes, such as visual skills [73] and perceived exertion [74]. More experienced elite athletes have been associated with more openness to experience and self-efficacy, which may, in turn, promote better self-confidence and use of coping skills [75].

Qualitative data reported that having more years of experience in futsal helped the athlete to absorb more psychological skills during the PSTMI intervention. This was evident during training sessions and competitive matches, where the newer/less experienced athletes often turned to the more experienced athletes for guidance, while the more experienced group of athletes served to motivate and encourage the less experienced group. However, despite their different levels of experience, all interviewed athletes reported psychological benefits from the sessions. This was in line with the quantitative data obtained.

### 4.3. Strengths and Limitations

Being the first known psychological and interventional mixed-methods study on futsal athletes, this addressed a clear research limitation in the literature [22], serving as a platform for further research. The mixed-methods approach capitalized on the strengths of both methods, compensating for each other’s weaknesses [76]. The quantitative component helped generalize the findings, while the qualitative component provided a deeper understanding [32]. Since futsal is generally sidelined due to the preferred financial interest in high-performance football, the researchers acknowledged this as a potential bias which might affect the generalizability of the findings. Therefore, the selected participants for this research were only registered with a futsal team, and not with a football team. Furthermore, when completing outcome measures, the participants were informed that their responses related to the chosen psychological variables should be specific to their futsal practice and competitive matches, and not to any other sport.

The chosen intervention (PSTMI) innovatively combined two well-researched interventions (PST and MI) to create a more inclusive approach. To minimize the Hawthorne effect [77], sessions were held without coaching staff, under strict confidentiality.

Inductive thematic analysis helped participants share their experiences with no preconceptions. Another strength was the close temporal proximity between the PSTMI and the knockout competition, simplifying skill implementation.

As for limitations, the uncontrolled nature of the study might have caused observation bias and type I error. This might have been partially resolved by using a mixed-methods design [78]. Another limitation included the small sample size used for quantitative analysis. However, this was a team intervention, and the whole team participated. No attrition occurred.

Knowledge ‘decay’ is common in pretest–posttest designs [79]. Reminder intervals helped retain information from previous sessions. Convenience sampling lacked generalizability [80]; however, most athletes had recently transferred from different teams, making the sample more representative.

Given that the outcome measures were collected at the start and at the end of the intervention, no outcome measures were collected in between sessions for the individual psychological skills (goal setting, motor imagery, arousal regulation, mindfulness, and self-talk). Therefore, this study was not able to measure the different impacts of the individual psychological skills. However, the aim of this study was to analyse the impact of the PSTMI (as one intervention) on the chosen psychological variables.

## 5. Conclusions

The PSTMI brought about medium-to-large improvements in the levels of mental toughness, cognitive anxiety, and coping skills in futsal players. Years of experience in futsal positively and significantly predicted athletes’ self-confidence and coping skills levels, minimizing competitive anxiety. The PSTMI was well-received by the athletes. Harboring a strong social identity towards the club was viewed as an asset. Therefore, this study revealed that good psychological preparation in futsal leads to better competitive performance and mental wellbeing. It also introduced a joint intervention (PSTMI) which offers a theoretical contribution to the literature.

Future recommendations include larger case-control studies, the dissemination of results to inform service development of the need for investment in sport psychology, and the formal inclusion of sport psychology topics in sports and coaching course material. The authors recommend such interventions to help athletes’ mental health, consequently improving their performance. Club management teams are encouraged to employ sport psychology practitioners, while the government and sport associations are encouraged to positively incentivize such appointments by offering financial grants to clubs who recruit sport psychology practitioners within the team. This will help create a sports culture where sport psychologist practitioners are a vital part of a team, just like the coaching staff, physiotherapist, and the rest of the medical team.

## Figures and Tables

**Table 1:** Paired Groups Including *t* Scores, *p*-Values and Effect Sizes (Hedge’s *g*).

		Mean (Pre)	Mean (Post)	Mean Difference	*t*	*df*	2-Sided *p*	Hedge’s *g*
Pair 1	SMTQ (Pre-Post)	37.85	41.85	−4.000	−2.996	12	0.011	0.778
Pair 2	CSAI-2-CA (Pre-Post)	23.85	21.00	−2.846	3.008	12	0.011	0.781
Pair 3	CSAI-2-SA (Pre-Post)	19.15	18.08	1.077	0.783	12	0.449	0.203
Pair 4	CSAI-2-SC (Pre-Post)	24.31	24.92	−0.615	−0.581	12	0.572	0.151
Pair 5	ACSI-28 (Pre-Post)	45.38	50.62	−5.231	−2.775	12	0.017	0.720

**Table 2:** Simple Linear Regression Analysing How Years of Experience Predicted Response Variables.

	Beta	*R*	*R* ^2^	*F*	*p*	*df (Res/Reg)*
SMTQ (Post)	0.405	0.405	0.164	2.157	0.170	1/11
CSAI-2-CA (Post)	−0.372	0.372	0.138	1.764	0.211	1/11
CSAI-2-SA (Post)	−0.477	0.477	0.228	3.242	0.099	1/11
CSAI-2-SC (Post)	0.648	0.648	0.420	7.971	0.017	1/11
ACSI-28 (Post)	0.646	0.646	0.418	7.897	0.017	1/11

## Data Availability

Data can be obtained from the corresponding author upon reasonable request.
